# Orthodontic treatment of patient with maxillofacial fibrous dysplasia: A case report

**DOI:** 10.4317/jced.55584

**Published:** 2019-03-01

**Authors:** Karthikeyan Subramani, Veronica Lee, Alex Krisko, Sarandeep Huja

**Affiliations:** 1 Roseman University of Health Sciences, College of Dental Medicine, Henderson, NV, USA; 2 Department of Oral and Maxillofacial Surgery, College of Dentistry, University of Kentucky, Lexington, KY, USA; 3 College of Dental Medicine, Medical University of South Carolina, Charleston SC, USA

## Abstract

Fibrous dysplasia is a benign skeletal disorder in which the normal bone and marrow are replaced by fibrous tissue and haphazardly distributed woven bone. The aim of this case report is to discuss the orthodontic treatment of a 13-year-old patient with fibrous dysplasia in the left maxilla. The patient had rotated maxillary second premolars, moderate crowding in both maxillary and mandibular arches with low maxillary frenal attachment. Orthodontic treatment was done with full fixed appliance and extraction of maxillary and mandibular third molars. Maxillary frenectomy and free gingival graft in mandibular anterior region were performed by a periodontist. The oral and maxillofacial surgery team monitored fibrous dysplasia in the left maxilla on a yearly interval. There is very limited information about orthodontic management of patients with craniofacial fibrous dysplasia. This case report discusses the orthodontic treatment and the importance of interdisciplinary approach in the management of patient with maxillofacial fibrous dysplasia.

** Key words:**Orthodontic treatment, fibrous dysplasia, maxillofacial fibrous dysplasia, case report.

## Introduction

Fibrous dysplasia (FD), first described by Lichtenstein in 1938, is a benign skeletal disorder in which the normal bone and marrow are replaced by fibrous tissue and haphazardly distributed woven bone (1). When it involves one bone, it is termed as Monostotic FD (MFD) and polyostotic FD (PFD) when it involves multiple bones. Patients with PFD may have McCune-Albright syndrome (MAS), which is the triad of PFD, café-au-lait skin macules, endocrinopathies and precocious puberty (2). Mutations in the α subunit of the stimulatory G protein encoded by the gene GNAS has been described as the etiology of FD (3).

Craniofacial bones, proximal femur and ribs are the most frequent and common locations of FD (4,5). Zygomatic-maxillary complex is reported to be the most commonly involved bone in MFD (6) and the craniofacial region is involved in almost 90% of cases of PFD (7). The signs and symptoms vary depending on the location of FD. If it involves the craniofacial bones, it can result in facial deformity, asymmetry and malocclusion. If the cranial base is involved, it can result in vision changes, hearing impairment, nasal obstruction, pain, or paresthesia (8). The most common characteristic of radiographic appearance is ‘ground glass’ of mixed radiolucency/opacity (9).

The differential diagnosis for craniofacial FD can be extensive. The patient’s history, growth characteristics of the lesion and radiographic examination are all important in narrowing the differential diagnosis. Other fibro-osseous lesions (ossifying fibroma, cemento-ossifying fibroma, cemento-osseous dysplasia, giant cell granuloma, and both aneurysmal and simple bone cysts) can be included in a broad differential diagnosis (8). Furthermore Paget’s disease, osteosarcoma and chronic sclerosing osteomyelitis should fall in the differential diagnosis for craniofacial FD (8,10). Rapidly changing, symptomatic, or extremely painful lesions require biopsy, if possible, as these may indicate a more aggressive FD, or a different serious diagnosis (3). Quiescent (unchanging) or asymptomatic lesions may not require biopsy with sufficient history, examination and radiography (3). In its quiescent or non-aggressive forms, FD is often discovered during routine dental examination or orthodontic evaluation. This case report discusses the orthodontic diagnosis and treatment of a patient with maxillofacial FD.

## Case Report

-History

A Caucasian female, 13 years 7 months of age, presented to the orthodontic clinic with chief complaint of “my canines are hurting and lower teeth are crooked.” A detailed dental, medical, and social history was obtained from the patient and patient’s parent. Patient had incipient secondary sex characteristics and had started menarche 2 years ago (her mother also had her menarche at the same age). The patient’s canine stage was H with apical foramen closed and cervical vertebral maturation (CVMS) stage 3 with 10-25% adolescent growth remaining. Her mother reported to have had similar malocclusion and patient reported of frequent headaches.

-Assessment

**Figure 1:**
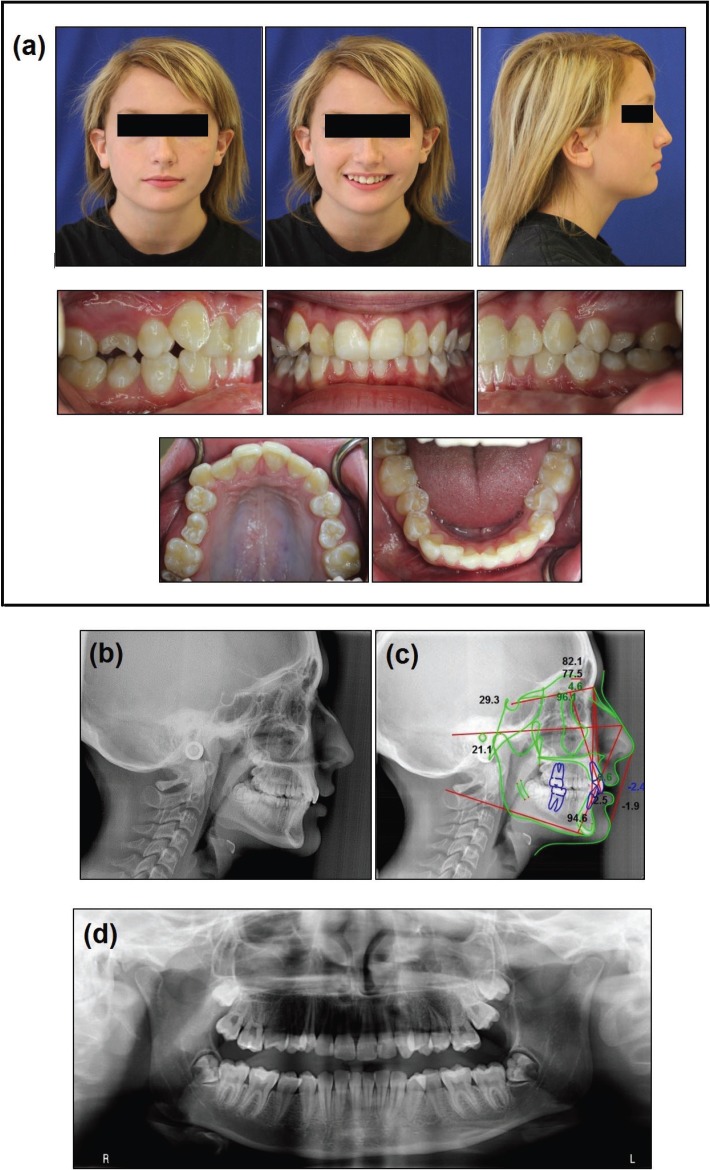
(a) Pretreatment facial and intraoral photographs, (b) Pretreatment cephalograph, (c) tracing, (d) panoramic radiograph showing opacification of left maxillary sinus region.

The clinical examination showed lip competence at repose, posterior divergent face with slightly increased facial convexity, obtuse nasolabial angle, decreased lower facial third, and retruded lower lip (Fig. 1). Patient had Class I molar and Class II canine relationship on right and Class III molar and Class II canine relationship on left with retrusive upper incisors and both overbite and overjet of 4mm. Maxillary and mandibular midline was on with MSP (midsagittal plane) and rotated maxillary second premolars were present in addition to 1.4mm and 5.2mm crowding in maxillary and mandibular arch. Posterior buccal crossbite of maxillary right second premolar was present and decreased attached gingiva in mandibular canine-canine region with low maxillary frenal attachment was observed (Fig. 1). The patient had generalized enamel decalcification and composite restoration on the mesial surfaces of maxillary lateral incisors (Fig. 1).

**Figure 2:**
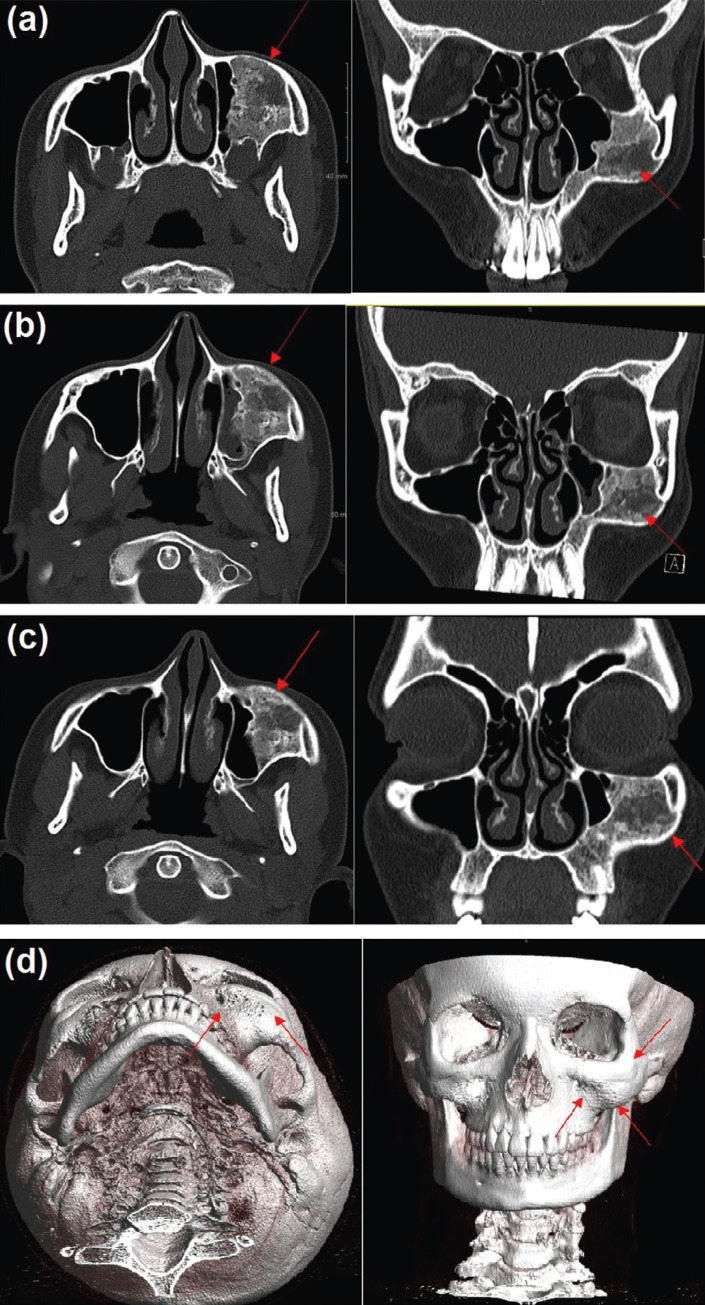
(a) Computed Tomography (CT) Face 2015: Axial and coronal views. Arrows indicating opacification of the left maxillary sinus and bony expansion of left maxilla. Note the “ground glass” appearance of the FD lesion indicated by the arrows, (b) CT Face 2016: Axial and coronal views. Note minimal change from previous scan taken in 2015, (c) CT Face 2017: Axial and coronal views. Minimal to no change in lesion size, (d) 3Dimensional reconstruction of Figure 2 (c). Arrows show size discrepancy between left and right maxilla due to expansion from FD.

The lateral cephalometric radiograph analysis revealed that patient was skeletal Class II with (ANB=4.6) due to a retrognathic mandible (SNB=77.5) and low mandibular plane angle (SN-MP=25.7) indicating hypodivergent growth pattern (Fig. 1c). The maxillary incisors were retroclined (U1-SN=95.1), and the mandibular incisors had slight proclination (IMPA=94.6). Patient had an obtuse nasolabial angle (110.9◦), and upper (-2.4mm) and lower (-1.9mm) lips were retrusive to the E-line (Fig. 1c). The panoramic radiograph showed cloudy left maxillary sinus (Fig. 1d). The treatment was initiated and the patient was referred to the oral and maxillofacial surgery department for extraction of maxillary and mandibular third molars. A CT scan was obtained for further evaluation of left maxillary sinus lesion. The scan showed an approximately 4 cm x 3 cm lesion in the left maxillary sinus with ground glass appearance in addition to mild bony expansion of the left maxilla (Fig. 2a). This lesion was consistent in appearance with fibrous dysplasia of the maxilla. The patient and family declined biopsy of the lesion; however, her history, specifically lack of endocrinopathies, combined with clinical and radiographic examination were sufficient for diagnosis of FD. At the time of diagnosis, the patient and family were entirely unaware of the lesion and mild facial asymmetry caused by the lesion. After diagnosis with FD, her stage of FD was thought to be either quiescent (no active growth), or non-aggressive (slow growth) (3). Close monitoring and repeat CT scans at yearly intervals were performed to further stage her FD.

-Treatment Objectives

The treatment objectives were to 1) resolve 1.4mm and 5.2mm crowding in the maxillary and mandibular arch and maintain transverse dimensions, 2) derotate maxillary second premolars, 3) correct posterior buccal crossbite of maxillary right second premolar, 4) maintain Class I molar on right, achieve Class I molar on left and Class I canine on both sides, 5) achieve optimum overbite and overjet, and 6) level the curve of spee and achieve ideal lip balance relative to E-plane.

-Treatment Alternative

The alternative treatment plan was to extract upper and lower second premolars to resolve crowding and to extract upper and lower third molars. After discussing with patient, non-extraction single-phase comprehensive treatment using full fixed appliance was chosen primarily due to lip support and profile concerns.

-Treatment Progress

Single-phase comprehensive non-extraction treatment (except for extraction of upper and lower third molars) using full fixed appliance was performed. The overall active treatment lasted 29 month followed by the delivery of upper Hawley retainer and lower bonded lingual retainer in 3-3 for retention.

-Following sequences of treatment was delivered:

0.022” pre-adjusted brackets (American Orthodontics Twin), Roth prescription were used. Oral hygiene instructions were reinforced to the patient throughout the treatment. 1) Bands were placed on the U/L first molars and bonded remaining teeth, placed lingual buttons on the U5s with the archwire (AW) sequence of 0.014” NiTi, 0.016” NiTi; 0.017” x 0.025” TMA. 2) Elastomeric power chain to derotate U5s. 3) Changed AWs to 0.017”x0.025” SS, protracted UL6 mesially. 4) Maxillary frenectomy and free gingival graft in mandibular anterior region were performed by a periodontist. 5) After sufficient healing period, 0.019”x0.025” SS mandibular AW with reverse curve of Spee was placed subsequently. 6) Bonded U7’s and 0.016” NiTi; 0.016”x0.022” NiTi as auxiliary AWs to align 7s. 7) Class II elastics from U3 to L6s to get canine in Class I relation. 8) Delivered upper Hawley and lower bonded lingual retainer in 3-3 after deband/debond.

CT scans taken at yearly intervals showed no change in lesion size (Fig. 2 (b,c,d).

-Treatment Results

**Figure 3:**
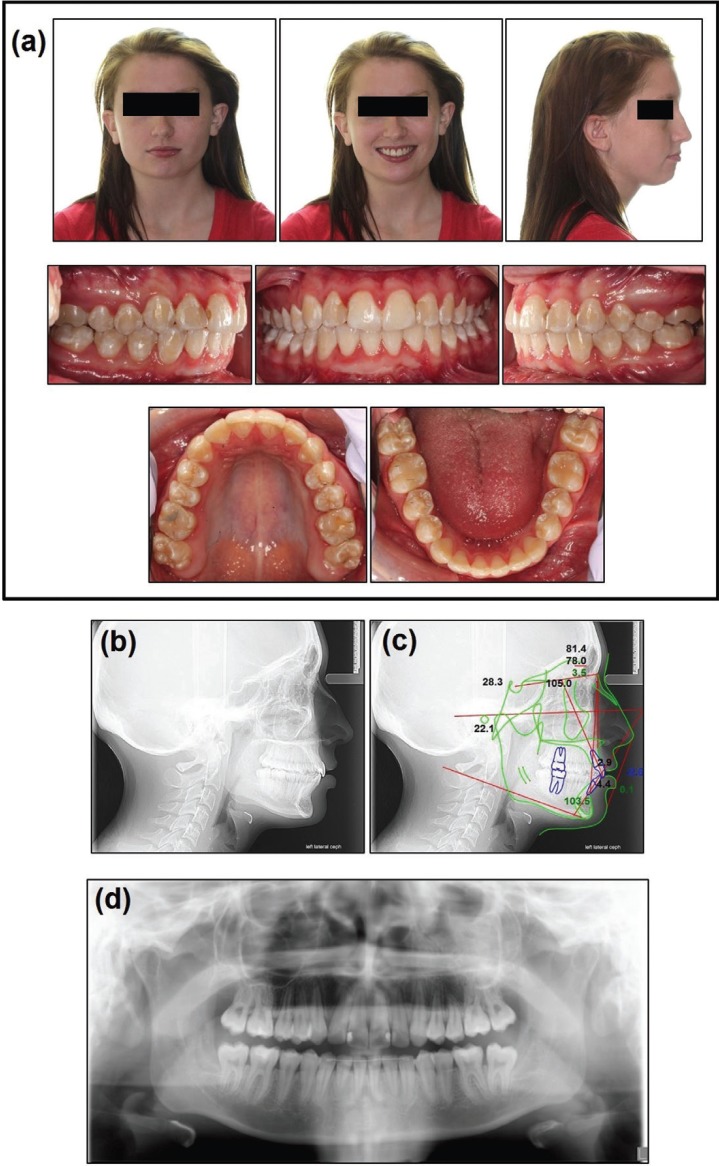
(a) Posttreatment facial and intraoral photographs, (b) Posttreatment cephalograph, (c) tracing and (d) panoramic radiograph.

The posttreatment records indicate that the treatment objectives were achieved. Both maxillary and mandibular crowding were resolved, maxillary second premolars were derotated and posterior crossbite of maxillary right second premolars was corrected (Fig. 3a). On the right side, Class I canine was achieved and the molar relation was maintained as Class I. On the left side, Class I canine and Class I molar relation was achieved. Curve of Spee was leveled and optimum overbite and overjet was obtained resulting in ideal lip balance relative to E-plane (Fig. 3c).

All three planes of maxilla were maintained since no orthopedics or surgery were performed and on the mandible with minimal downward growth and backward rotation. On the maxillary dentition, incisors were proclined (U1-SN, from 95.1° to 105.1°). Fair vertical control of maxilla was observed with the maxillary incisors extruded slightly and intermolar width reduced by 1.8mm. On the mandibular dentition, the lower incisors were proclined (IMPA, from 94.6°-103.5°), overbite was reduced from 4mm to 2mm, intermolar and intercanine width were reduced by 1.1mm and 1.5 respectively. In terms of facial esthetics, no significant facial change was noticed. No significant facial asymmetry was noticed from the FD.

## Discussion

Approximately 90% of patients with FD have lesions in craniofacial bone. This includes maxilla and mandible and these lesions can grow rapidly to cause expansion of bone and displace orbit and teeth resulting in severe malocclusion and facial disfigurement (7,9,11,12). Maxillo-mandibular FD is also associated with dental development disorder from metabolic dysfunction and disordered bone architecture that affect tooth development and eruption (13,14). The most common dental anomalies associated with FD include tooth rotation, oligodontia, displacement, enamel hypoplasia, enamel hypomineralization, taurodontism, retained deciduous teeth and attrition (15). Our patient had rotation of maxillary second premolars (U5s) and crowding/malocclusion, which can be attributed to FD.

High caries index is indicated in patients with maxillo-mandibular FD, attributed to the severe malocclusion, increased enamel hypoplasia and hypomineralization (11). Due to severe malocclusion and high caries index, more frequent dental recall is recommended to control dental plaque accumulation. Our patient had fair oral hygiene throughout the treatment with high caries index from malocclusion and generalized white discoloration with discolored composite restoration in mesial surface of U2s, which were recommended for future restorative consult for better esthetics.

FD in the craniofacial skeleton, causing significant dysmorphic features such as facial asymmetry and deformity and dental anomalies have radiographic appearance of radiolucency/opacity of ground glass morphology. Our patient was diagnosed with FD as evidenced by panoramic radiographs and CT scans in the left maxillary sinus area, which was significantly filled with FD bone tissue and radiopacity (Fig. 1d). This homogenous feature is most common during childhood and adolescence, then the lesions become less radiolucent, more mixed, and heterogeneous with age (9,11). Taurodontism seen on the dental radiographs may be associated with endocrine disorders, such as growth hormone excess, and can be an indicator of an underlying endocrinopathy associated with MAS (11,16,17).

One of most common symptom of FD is pain and adults are more likely to have more severe pain than children, suggesting that there is an age-related increase in the prevalence of pain (18). Bisphosphonates have been moderately effective in pain management, but no effect has been found to alter the disease course (19). More recently Denosumab, a monoclonal antibody to Receptor activator of nuclear factor kappa-Β ligand (RANKL), has been explored as a medical treatment for FD resulting in the reduction in bone pain, tumor growth, and bone turnover markers (20). Another study has examined Tocilizumab, a monoclonal antibody to Interleukin-6 (IL-6), and have found it may be effective in treating bone pain refractory to bisphosphonate therapy (21). Regardless, there are still no accepted medical treatments for curing or stopping the progression of the disease (19-21). One of our patient’s chief concern was pain in the canine area and this can be due to the FD, which is located in the left maxillary sinus, and pressure can attribute to the pain in the area.

Surgical treatment for patients with craniofacial FD should be individualized to each patient. If patients are satisfied with their appearance and their FD is quiescent or non-aggressive, watchful waiting is deemed to be an acceptable form of treatment (3). In patients with visual disturbance or patients who are unsatisfied with their appearance, surgery is the most accepted treatment modality (22). Certain authors recommend conservative therapy, which involves recontouring the bone to restore normal bony shape (22). Advantages of this therapy is that it is relatively non-invasive and is easily accepted by patients; however need for re-operation is higher with conservative treatment (23). Other surgical groups lean towards radical excision of the lesion with immediate reconstruction, if possible.(6) This method has a lower incidence of re-operation and has curative potential; however, this method does have increased comorbidity due to the more extensive surgery (23). Our patient was monitored by yearly CT scan for two years after the initial diagnosis and her FD appeared to be in a quiescent stage (Fig. 2b,c). The patient and family elected to forego surgery and are following up at regular intervals for monitoring.

Reports in the literature so far indicate that patients with FD do not require special dental management and were able to undergo routine dental care safely and successfully without exacerbation and complication of FD lesions (3,24). The reported procedures included dental restoration, tooth extraction, orthodontic therapy, odontoma removal, maxillary cyst removal and biopsy of jaws (16). However, due to having access to very few and smaller patient pool, the limited data on effectiveness and outcome of dental procedures in maxillo-mandibular FD/MAS patients of current clinical challenges faced by dental anomalies of FD/MAS patient is unclear (25). In our case, patient preferred not to do any extractions for the orthodontic treatment and her treatment did not have any effect on her FD lesion even after extracting all third molars during the treatment.

In addition, literature reported among the patients that received orthodontic therapy, the duration of treatment appeared to take longer than normal (2-4 years) and the results were less satisfactory and there was relapse (3,16). One theory reported more rapid orthodontic tooth movement in FD involved bone but, data to support this is currently lacking (8). The duration of treatment for our patient was within normal time frame and no significant delay in treatment was found in relevance to the FD and satisfactory results were delivered for the orthodontic treatment and maxillary Hawley retainer and lower lingual bonding retainers on 3-3 were delivered to prevent relapse.

It is advised to delay orthodontic therapy till after age of skeletal maturity based on patients’ needs and outcomes of orthodontic evaluation since FD disease activity decreases after skeletal maturity (8). Our patient initiated treatment at CVMS 3 (Fig. 1b) when peak in mandibular growth has already occurred and because of possible relapse on the U5s after orthodontic treatment, patient was referred to periodontist for circumferential supracrestal fibrotomy procedure for prevention of relapse.

## Conclusions

The goal of management in craniofacial FD is to mainly focus on the improvement of function and esthetics. In this report, the patient diagnosed with FD had successful orthodontic treatment. For, MAS/FD, the dental professional must be able to recognize characteristic signs of FD and be able to work in a team with other medical colleagues to develop appropriate treatment strategies.
